# A divergent spirochete strain isolated from a resident of the southeastern United States was identified by multilocus sequence typing as *Borrelia bissettii.*

**DOI:** 10.1186/s13071-016-1353-4

**Published:** 2016-02-04

**Authors:** Maryna Golovchenko, Marie Vancová, Kerry Clark, James H. Oliver, Libor Grubhoffer, Nataliia Rudenko

**Affiliations:** Biology Centre Czech Academy of Sciences, Institute of Parasitology, Ceske Budejovice, 37005 Czech Republic; The James H. Oliver Jr. Institute for Coastal Plain Sciences, Statesboro, Georgia Southern University, Georgia, 30460-8056 USA; University of North Florida, Jacksonville, FL 32224 USA; University of South Bohemia, Ceske Budejovice, 37005 Czech Republic

**Keywords:** *Borrelia*, *Borrelia bissettii*, MLST analysis, live spirochete, divergent strain

## Abstract

**Background:**

Out of 20 spirochete species from *Borrelia burgdorferi* sensu lato (s.l.) complex recognized to date some are considered to have a limited distribution, while others are worldwide dispersed. Among those are *Borrelia burgdorferi* sensu stricto (s.s.) and *Borrelia bissettii* which are distributed both in North America and in Europe. While *B. burgdorferi* s.s. is recognized as a cause of Lyme borreliosis worldwide, involvement of *B. bissettii* in human Lyme disease was not so definite yet.

**Findings:**

Multilocus sequence typing of spirochete isolates originating from residents of Georgia and Florida, USA, revealed the presence of two *Borrelia burgdorferi* sensu stricto strains highly similar to those from endemic Lyme borreliosis regions of the northeastern United States, and an unusual strain that differed from any previously described in Europe or North America. Based on phylogenetic analysis of eight chromosomally located housekeeping genes divergent strain clustered between *Borrelia bissettii* and *Borrelia carolinensis*, two species from the *B.burgdorferi* s.l. complex, widely distributed among the multiple hosts and vector ticks in the southeastern United States. The genetic distance analysis showed a close relationship of the diverged strain to *B. bissettii*.

**Conclusions:**

Here, we present the analysis of the first North American human originated live spirochete strain that revealed close relatedness to *B. bissettii*. The potential of *B. bissettii* to cause human disease, even if it is infrequent, is of importance for clinicians due to the extensive range of its geographic distribution.

## Background

The *Borrelia burgdorferi* sensu lato (s.l.) complex is a diverse group of bacteria widely distributed in temperate regions of the Northern Hemisphere. From the time of the discovery of the causative agent of Lyme borreliosis (LB), a large number of *Borrelia* isolates has been obtained from various vertebrate species, including humans. To date, five of the genospecies from *B. burgdorferi* s.l. complex are ensured human pathogenic, including *B. burgdorferi* sensu stricto, *B. afzelii*, *B. garinii*, *B. bavariensis*, and *B. spielmanii*. Occasionally, *B. lusitaniae* and *B. kurtenbachii* have been detected in patients [1–7]. The recognition of *B. bissettii* as a causative agent of human LB to date was supported by rare cases of molecular detection of *B. bissettii* DNA in samples of human origin [8–10]. Detection of sequences highly similar to *B. bissettii* strain DN127 in cardiac valve tissue of a patient with endocarditis and aortic valve stenosis [8], and in serum samples of patients in the Czech Republic [9], raised concern about the pathogenic potential of *B. bissettii*. More recently, the first molecular evidence of infection of 3 out of 27 (11 %) studied residents of Mendocino County (California, USA) with a *B. bissettii*-like spirochete highly similar to strain DN127 further suggested that this species is able to infect humans although LB symptoms were only reported in individuals that had concurrent *B. burgdorferi* infections [10].

## Findings

### Methods

#### General analysis of Borrelia isolates

Total DNA was purified from pelleted spirochetes of 3rd passage, cultured from plasma of three residents of southeastern USA [11] in modified Kelly-Pettenkofer (MKP) media [12]. Plasma samples (samples M6p and M11p from Georgia; sample M7p from Florida) were collected earlier for microbiologic testing as part of ongoing studies of tick-borne diseases in the southern United States (UNF IRB approval #468310-3), but not for the purpose of this study. Written informed consent was obtained from each patient prior to enrolment into the study.

DNA purification, sequencing and sequence analysis were conducted according to protocols established in our laboratory [11, 13]. MLST analysis was conducted according to the scheme developed by Margos and colleagues [14]. Briefly, total DNA from cultured spirochetes was purified using the DNeasy Blood and Tissue kit strictly according to manufacturer’s protocol (Qiagen, USA). The Master*Taq* Kit (Eppendorf, Germany) with 5x *Taq*Master® PCR enhancer was used for amplification of eight housekeeping genes using previously described nested primers for *clpA, clpX, pepX, recG, rplB* and *uvrA* and semi-nested primers for *nifS* and *rplB* under PCR conditions described earlier [14]. The purified PCR products of expected size were submitted for direct sequencing to the Genomic Laboratory of the Biology Centre Czech Academy of Sciences (Ceske Budejovice, Czech Republic). Sequencing was conducted in both directions, using the same primers that were used for amplification of each locus.

#### Phylogenetic analysis

Multiple alignments of the concatenated sequences of the genes *clpA, clpX, nifS, pepX, pyrG, recG, rplB* and *uvrA* were generated in MEGA5 [15]. Sequences of 20 LB group species were downloaded from the MLST database (http://pubmlst.org/borrelia/). A maximum likelihood tree was constructed in MEGA5 using default settings and 500 bootstrap repeats. The general time reversible model was selected. A genetic distance analysis was conducted in MEGA5 using the Kimura 2-parameter model [16] and the concatenated sequences of housekeeping loci. The rate variation among sites was modelled with a gamma distribution (shape parameter = 0.6). The analysis involved 29 nucleotide sequences (four *B. carolinensis*, one *B. americana*, one *B. kurtenbachii*, thirteen *B. bissettii* and seven *B. burgdorferi* sequence types (STs) and the three strains analysed in this study). Codon positions included were 1st + 2nd + 3rd + Noncoding. All positions containing gaps and missing data were eliminated. There were a total of 4785 positions in the final dataset. The evolutionary analysis was conducted in MEGA6 [17].

### Results

#### Multilocus sequence typing and phylogenetic analysis

MLST of spirochete isolates originating from residents of Georgia and Florida, USA, revealed the presence of two *B. burgdorferi* sensu stricto strains, named M6p and M11p, highly similar to those from the northeastern United States, and an unusual strain, M7p, that differed from any previously described in Europe or North America.

**Fig. 1:**
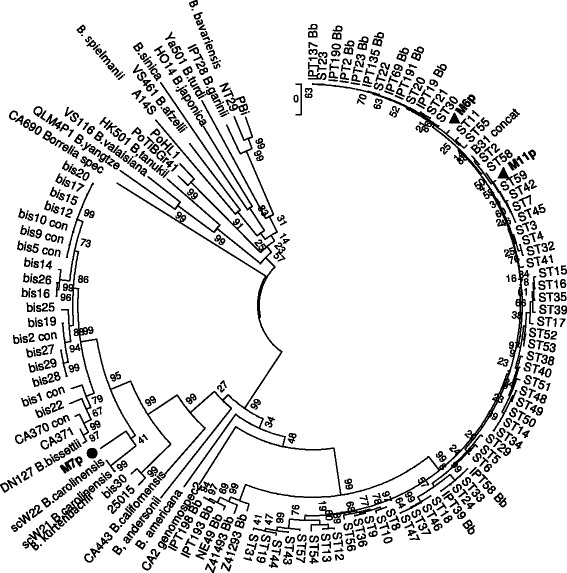
A maximum likelihood tree was generated in MEGA5 [15] using concatenated sequences of eight housekeeping loci (*clpA, clpX, nifS, pepX, pyrG, recG, rplB* and *uvrA*). The general time reversible model was employed and 500 bootstrap repeats used for estimation of node support. The large clade on the right hand site represents *B. burgdorferi* s.s. and two of the samples analyzed in this study (M6p and M11p, marked with ▲) fall into this clade. The third strain analyzed (M7p, ●) clusters between *B. carolinensis* and *B. bissettii*

Results of MLST analysis showed that samples M6p and M11p (indicated by ▲, Fig. 1) clustered within the *B. burgdorferi* s.s. clade (Fig. 1). Strain M6p was found close to ST11, and strain M11p was positioned between ST58 and ST59. The affiliation of these samples to the species *B. burgdorferi* s.s. was confirmed by a genetic distance analysis (Table 1); the values for genetic distance were very low (<=0.004) to all *B. burgdorferi* STs included in the analysis, even those that did not represent next neighbors in the tree, e.g. B31, ST23, ST22. The number of variable sites to the closest neighbors in the tree within the 4785 nucleotides, i.e. ST11 and ST30 for M6p and ST58 and ST59 for M11p, was 17 and 12 and eight and seven, respectively.

**Table 1:**
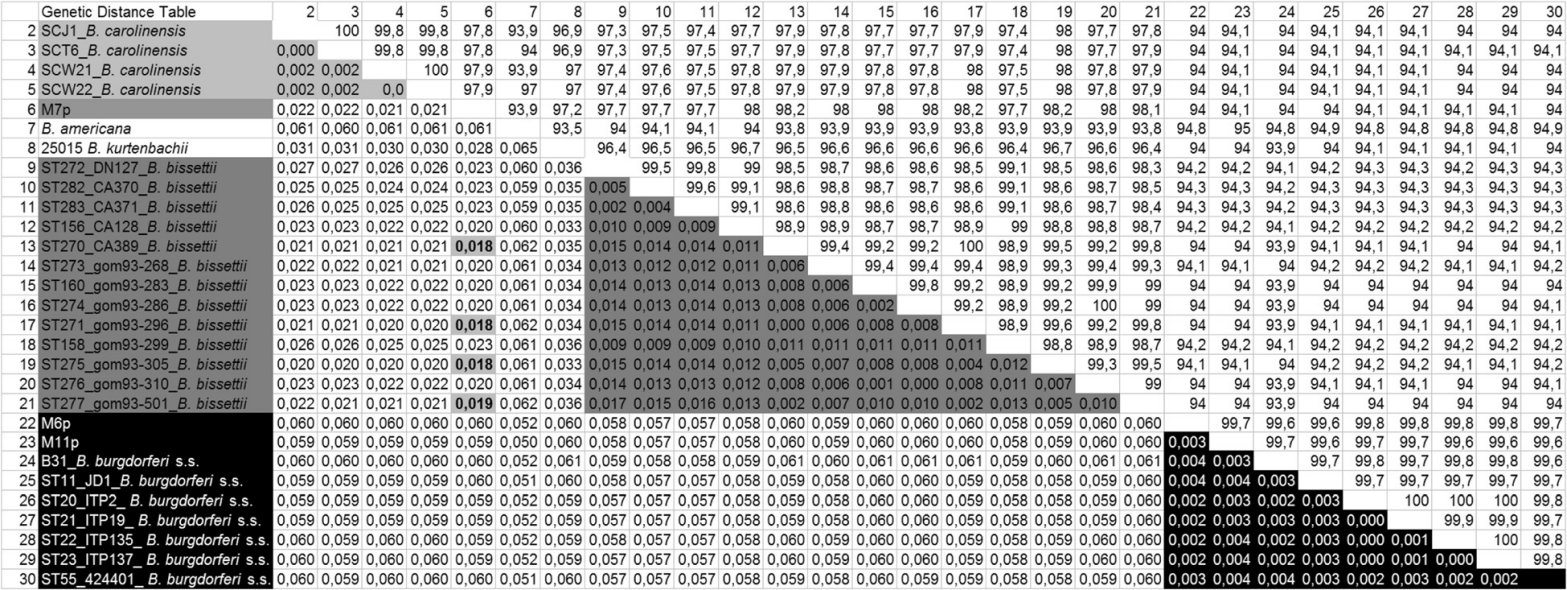
Estimates of evolutionary divergence between sequences

The numbers of base substitutions per site between sequences are shown in the lower left panel. In the upper right genetic similarities values are given. Strain M7p showed values that just exceeded the species threshold compared to some *B. bissettii* strains (bold numbers and shaded in light grey in column 6). The shaded regions in columns 2–4, 9–20 and 22–30 correspond to intraspecific divergence within genospecies (*B. carolinensis*, *B. bissettii* and *B. burgdorferi* sensu stricto, respectively). Analyses involved 29 nucleotide sequences and were conducted using the Kimura 2-parameter model [16]. Codon positions included were 1st + 2nd + 3rd + Noncoding. All positions containing gaps and missing data were eliminated. There were a total of 4785 positions in the final dataset. Evolutionary analyses were conducted in MEGA6 [17]

Based on phylogenetic analysis the third sample, M7p (indicated by ●, Fig. 1), did not fall into the clade of a given species but clustered between *B. carolinensis* and *B. bissettii,* two species widely distributed among the multiple hosts and vector ticks in the southeastern United States, although the node support was low (41 %). The genetic distance analysis showed a value above the species threshold (0.017) compared with four *B. carolinensis* strains (0.021) and the majority of *B. bissettii* STs (0.022) (Table 1). However, for four *B. bissettii* STs (ST270 from California; ST271, ST275 and ST277 from Colorado) the genetic distance was just above the species threshold (0.018 and 0.019; species threshold 0.017) suggesting a close relationship of the M7p strain to *B. bissettii.* The species threshold of 0.017 for the MLSA System based on housekeeping genes was determined by comparison to data published earlier [18].

#### Nucleotide sequence accession numbers

Sequences determined in this study have been deposited into GenBank and given the indicated accession numbers (numbers for each isolate are given for sequenced genomic loci in the following order: (*clpA, clpX, nifS, pepX, pyrG, recG, rplB* and *uvrA*) KM269425, KM269422, KM269428, KM269434, KM269443, KM269440, KM269437, KM269431 for M6p; KM269426, KM269423, KM269429, KM269435, KM269444, KM269441, KM269438, KM269432 for M11p; KM269427, KM269424, KM269430, KM269436, KM269445, KM269442, KM269439, KM269433 for M7p.

### Discussion

In this study we investigated *Borrelia* strains that were isolated from residents in Southeastern US including one that was closely related to *Borrelia bissettii*. This is the first report of a *B. bissettii* being isolated from a human individual in the US. Until this research only *B. burgdorferi* s.s. strains were identified in samples of human origin in North America [10]. Today the number of reports that associate *B. bissettii* with human LB can be counted on one hand [8–10]. The lack of clinical evidence does not allow association of this spirochete species with specific clinical manifestations of the disease. However, earlier findings suggest that arthralgia is one of the disease manifestations caused by infection with *B. bissettii* in a group of LB diagnosed patients [9]. A single case of human endocarditis confirmed a known fact that LB spirochetes in general and *B. bissettii* in particular have a predilection for cardiac infection [8]. Those data are well correlated with a study of pathogenic potential of *B. bissettii* on murine model. The ability of *B. bissettii* strains to induce lesions in vertebrate host heart, femorotibial joint and bladder was confirmed 8 weeks after laboratory infection of mice with spirochete isolates [19].

Strains of *B. bissettii* reported thus far in the United States occur in rather moderate climatic regions of the western and southeastern parts of the country, but rarely in the northern region. *B. bissettii* is maintained in several enzootic transmission cycles that involve both human-biting “bridge” vectors (*I. pacificus*) and ticks with a strong host preference that do not bite humans (*I. spinipalpis*) in the west [20] and in the east coast (*I. minor, I. affinis*) [21]. *B. bissettii* strains were isolated from the dusky-footed wood rat (*Neotoma fuscipes*) and *I. spinipalpis* from California [22], and from the cotton mouse, the cotton rat, the eastern wood rat, *I. scapularis*, *I. minor* and *I. affinis* ticks from South Carolina and Georgia [23], [Oliver Jr., Rudenko, Golovchenko and Clark, unpublished observations]. *B. bissettii* was detected in co-infected isolates originated from skin biopsies of downy woodpecker *(Dryobates pubescens)*, Carolina wren (*Thryothorus ludovicianus*) and northern water thrush (*Parkesia noveboracensis*), trapped in St. Catherines Island (GA, USA) in 1997 [Oliver Jr., Golovchenko and Rudenko, unpublished observations]. The records of naturally occurring infections in multiple species of rodents, birds, and *Ixodes* ticks indicate that *B. bissettii* is widely distributed in the United States and is not narrowly confined to particular vertebrate host or tick species. With such well-established population of *B. bissettii* in its area of distribution, with wide availability of multiple hosts and tick vectors, it is not surprising that residents of the west and east coast are exposed to this species.

The pathogenic potential of American strains of *B. bissettii* investigated on murine models was sufficient to induce disease, causing pathology within the heart, femorotibial joint and bladder in vertebrate host [19]. At the same time, European studies had shown that *B. bissettii* are able to infect humans and suggested they may be potentially human pathogenic [8, 9] although the published cases of human infections may be infrequent due to the rarity of *B. bissettii*-infections in human-biting host seeking *Ixodes* vectors [23–27].

Here, we present analysis of the first recovered live *B. bissettii*-related spirochete (strain M7p) from a resident of North America. The taxonomic status of this strain requires further investigation. Phylogenetic analysis resulted in clustering of M7p strain between two clades formed by *B. carolinensis* and *B. bissettii* with rather low node support (41 %). The value of genetic distance was above the species threshold compared with *B. carolinensis* and *B. bissettii*, which might mean that strain M7p represents a novel species from *B. burgdorferi* s. l. complex. The species threshold of 0.017 for the MLSA based on housekeeping genes was determined by comparison to data published by Postic and colleagues earlier [18]. These authors compared DNA-DNA hybridization data with MLST data and used strains Z41293 and NE49 to set the threshold for species delineation in *Borrelia*. The same strains were used later [28] in the MLSA system based on housekeeping loci which allowed direct comparison of genetic distances between MLSA systems. It was found that strain NE49 that belongs to the same clade as Z41293 has a slightly different distance of 0.0183. Thus, species thresholds are not determined by a strictly “fixed” genetic distance but maybe a little bit fuzzy. Another confirmation of this fact was present in a recent report about *B.yangtzensis* [29]. Two quite divergent populations of *B. yangtzensis* do exist with genetic distances above the species threshold [28]. These two divergent populations are “bridged” by a population that has genetic distances below the species threshold to both of these divergent populations, making this an example for a genetic continuum between divergent populations of a species. To analyze more strains similar to M7p described in this study is very important to get a better picture how *Borrelia* species and populations are structured.

Isolation of live *B. bissettii*-like spirochetes from a human provides evidence that this species, in addition to *B. burgdorferi* sensu stricto, may cause human Lyme borreliosis in North America, possibly with clinical manifestations different from those related to *B. burgdorferi* s.s. infection [30].
